# Is right coronary artery chronic total vessel occlusion impacting the surgical revascularization results of patients with multivessel disease? A retrospective study

**DOI:** 10.7717/peerj.4909

**Published:** 2018-06-15

**Authors:** Janusz Konstanty-Kalandyk, Krzysztof Bartuś, Jacek Piątek, Anna Kędziora, Tomasz Darocha, Krzysztof L. Bryniarski, Marcin Wróżek, Piotr Ceranowicz, Stanisław Bartuś, Leszek Bryniarski, Bogusław Kapelak

**Affiliations:** 1 Department of Cardiovascular Surgery and Transplantology, Institute of Cardiology, Jagiellonian University Medical College, The John Paul II Hospital, Krakow, Poland; 2 Department of Interventional Cardiology, Institute of Cardiology, Jagiellonian University Medical College, The John Paul II Hospital, Krakow, Poland; 3 Department of Physiology, Jagiellonian University Medical College, Krakow, Poland; 4 2nd Department of Cardiology, Jagiellonian University Medical College, The University Hospital, Krakow, Poland; 5 1st Department of Cardiology, Interventional Electrocardiology, and Arterial Hypertension, Jagiellonian University Medical College, The University Hospital, Krakow, Poland

**Keywords:** Coronary artery disease, Chronic total occlusion, Surgical revascularization, Short-term outcome, CABG, MACE

## Abstract

**Introduction:**

Chronic total occlusion (CTO) is common in the presence of other significantly narrowed coronary arteries. The impact of total occlusion and its association with completeness of revascularization on patients with multivessel disease undergoing coronary artery bypass graft (CABG) remains largely unknown.

**Aim:**

The aim of our study was to compare CABG operation characteristics, as well as 30-day mortality, incidence of post-operative major adverse cardiac and cerebrovascular events (MACCE) between patients with and without CTO in right coronary artery (RCA).

**Materials and Methods:**

A total of 156 consecutive patients were included in the analysis. CTO of RCA or right posterior descending artery (RPD) was diagnosed in 57 patients (CTO-RCA group). Coronary stenosis without CTO in RCA was diagnosed in 99 patients (nonCTO-RCA group). Baseline characteristics were comparable in both groups.

**Results:**

The majority of patients had class II (49.1% vs. 46%, *p* = 0.86) or class III (42.1% vs. 43%, *p* = 1.0) Canadian Cardiovascular Society grading system symptoms. Patients in the CTO-RCA group had in average 2.2 grafts implanted, as opposed to 2.4 grafts in patients in the nonCTO-RCA group (*p* = 0.003). Graft to the RCA was performed in 40.3% patients in the CTO-RCA group and in 81% patients in the nonCTO-RCA group (*p* = 0.001). The 30-day mortality from any cause or cardiac cause did not differ between groups (7% vs. 2%, *p* = 0.14 and 3.5% vs. 2%, *p* = 0.57 respectively). In a multivariate analysis CTO in RCA or RPD and peripheral artery disease were independent predictors of post-operative MACCE (7.9 (1.434–43.045) *p* = 0.02; 18.8 (3.451–101.833) *p* < 0.01, respectively).

**Conclusions:**

Chronic total occlusion of RCA was found to be associated with smaller number of grafts performed during the CABG procedure. Although mortality between patients in the CTO-RCA and nonCTO-RCA groups did not differ, patients in the CTO-RCA group had higher incidence of post-operative MACCE.

## Introduction

Coronary chronic total occlusion (CTO) is a distinct subset of coronary artery disease (CAD) and it is defined as a coronary artery with absent anterograde blood flow for more than 3 months. In patients with multivessel CTO, surgical revascularization of the CTO arteries varies, ranging from 69% to 89% depending on the study (Pereg et al., 2016; Campeau et al., 1984; Cashin et al., 1984). For patients undergoing revascularization due to the CTO, coronary artery bypass grafting (CABG) (as opposed to percutaneous coronary intervention (PCI)) is performed in nearly 75% of the cases (Strauss, Shuvy & Wijeysundera, 2014; Fefer et al., 2012).

Most CTOs are successfully bypassed, however revascularization failures are more frequently observed in CTOs in areas other than the left anterior descending artery (LAD) (Fefer et al., 2012; Widimsky et al., 2004). Coronary arteries other than LAD with CTOs targeted for bypass are often atherosclerotic, narrow with diffuse disease or heavy calcification. Moreover, high vascular resistance in CTOs results in poor blood runoff from the bypass graft, and contributes to graft failure (Fefer et al., 2014).

In our study, we analyzed clinical outcomes of patients undergoing CABG in order to assess the prevalence of coronary CTO in right coronary artery (RCA) in patients with 3-vessel coronary disease, rate of success in grafting coronary arteries with CTO-RCA and impact of CTO-RCA on clinical outcomes during the first 30 post-operative days.

## Materials and Methods

Between January 2015 and July 2015, clinical and angiographic data of 156 consecutive patients was retrospectively analyzed. All patients had 3-vessel disease and were scheduled for elective surgery. Preoperative coronary angiograms were reviewed in detail by two cardiologists blinded to subsequent clinical outcomes. In all of the patients RCA was a dominant coronary artery. All patients were qualified for the revascularization based on a Heart Team decision after confirming myocardial viability with echocardiography or additional imaging if necessary. In all patients the severity of mitral regurgitation was assessed to be trivial or mild. Patients with moderate or severe mitral regurgitation, in whom repair or replace mitral valve was considered, were not included in the study group.

Hypokinesis of posterior heart wall was observed in all patients. It should be emphasized that clearly all patients who presented with CTO-RCA/right posterior descending artery (RPD) underwent myocardial ischemia in the area of the RCA blood supply. A large part of these patients, however, did not have any symptoms of sudden myocardial ischemia (silent ischemia).

The surgeries were performed following standard CABG protocol with left internal thoracic artery to LAD whenever possible and vein grafts to other narrowed arteries. For graft to RCA/RPD, vein was used in 96% cases in nonCTO-RCA group and in all cases in CTO-RCA group. In terms of CTO a graft is placed in the most proximal part of the vessel after the occlusion, depending on the technical possibilities to perform anastomosis.

Post-operative outcomes were defined in accordance to previous reports (Banerjee et al., 2012) and as it is routinely evaluated in reporting in adult cardiac surgery. Major adverse cardiac and cerebrovascular events (MACCE) were defined as the composite of death, myocardial infarction (MI), repeat coronary revascularization, and stroke. MI was defined as a presentation with chest pain and/or ischemic electrocardiographic changes and/or shock, along with elevated levels of cardiac biomarkers (at least twice the upper limit of normal) (Thygesen et al., 2012). Cardiac biomarkers were collected three times in early (48 h) post-operative period. Hypertension was defined as intake of antihypertensive medications, hyperlipidemia as intake of lipid lowering therapy, and diabetes mellitus as intake of insulin or hypoglycemic medications. Chronic kidney disease (CKD) was defined as glomerular filtration rate <60 mL/min/1.73 m^2^ for more than 3 months or a discharge diagnosis of CKD.

Chronic total occlusion in LAD was grouped as CTO in LAD distribution. Similarly, CTO in the left circumflex (LCX) and major obtuse marginal branches were classified as LCX distribution CTO. CTO in the RCA or RPD were classified as RCA territory CTO.

We have divided patients into two groups: CTO-RCA group (CTO in RCA or RPD) and nonCTO-RCA group (without CTO in RCA).

Successful bypass grafting of an epicardial coronary vessel was determined based on the CABG operative report.

The primary study outcome was 30-day all-cause mortality, cardiac mortality, MI and incidence of MACCE. The secondary outcomes were defined as successful grafting of epicardial coronary arteries with CTO and occurrence of adverse events (atrial fibrillation, sternal wound complication and major bleeding).

The study was approved by the Bioethics Committee of Krakow Regional Medical Chamber (L.dz.OIL/KBL/OIL/11/216). Written consent was obtained from all patients.

### Statistical analysis

Statistical analysis was performed using Statistica 12.0 software. Results were presented based on the parameters of descriptive statistics, including mean values and its standard deviations, or median values and its quartiles, as appropriate. Categorical variables were presented as percentages. Continuous variables were compared via Student’s *t*-test and categorical variables via Chi-square test. Univariate and multivariate stepwise logistic regression were used to determine risk factors for MACCE. *P* value less than 0.05 was considered significant.

The created model is standardized for age, gender, left ventricle function (assessed by left ventricle ejection fraction), previous MI and previous stroke. The power of the study was calculated on the level of 0.80 (80 percent).

## Aim

The aim of our study was to compare CABG operation characteristics and incidence of MACCE during the first 30 post-operative days between patients with and without CTO in RCA.

## Results

Overall 156 patients were analyzed. CTO in RCA was present in the preoperative angiogram of 57 (36.5%) patients. Baseline characteristics were comparable between CTO-RCA and nonCTO-RCA groups (Table 1). The median age of the patients in CTO-RCA groups was 67 ± 9.6 years, whereas in nonCTO-RCA group it was 67 ± 9.4. Likewise, median left ventricular ejection fraction did not differ between patients in CTO-RCA and nonCTO-RCA groups (53 ± 12 vs. 55 ± 11.1) and most of the patients in both groups presented with Canadian Cardiovascular Society (CCS) class II or III symptoms.

**Table 1:** Clinical and operative characteristics at baseline.

	CTO RCA/RPD (*N* = 57)	Non-CTO RCA/RPD (*N* = 99)	*p* value
Age—yr	67 ± 9	66 ± 9	0.95
Female sex—no. (%)	40 (70)	76 (76)	0.45
Documented peripheral artery disease—no. (%)	7 (12.2)	7 (7)	0.38
Hypertension treated with drugs—no. (%)	52 (91.7)	91 (92)	1.0
Diabetes—no. (%)	22 (38.6)	31 (31)	0.38
Documented atrial fibrillation—no. (%)	5 (8.7)	8 (8)	1.0
Previous stroke—no. (%)	7 (12.3)	7 (7)	0.38
Documented myocardial infarction—no. (%)	34 (60)	45 (45)	0.06
EF mean (%)	53.4	54.5	0.8
Mitral regurgitation: mild—no. (%)	35 (61)	61 (62)	0.9
Trivial—no. (%)	22 (39)	39 (38)	–
**Coronary artery stenosis**			
Left main coronary artery disease—no. (%)	21 (36.8)	46 (46.9)	0.24
CTO RCA/RPD—no. (%)	57 (100)	0 (0)	–
CTO LAD—no. (%)	7 (12.3)	11 (11)	0.8
CTO Cx/Mg—no. (%)	11 (19.3)	12 (14)	0.49
**Symptoms of coronary artery disease**			
CCS angina class—no. (%)			
I	0	0	
II	28 (49.1)	46 (46)	0.86
III	24 (42.1)	43 (43)	1.0
IV	5 (8.8)	11 (11)	0.78
**Operative data**			
Graft per patient—no.	2.1	2.4	0.003
RCA/RPD graft—no. (%)	23 (40.3)	81 (81)	0.001

**Notes:**

Plus–minus values are mean ± SD. Canadian Cardiovascular Society (CCS) angina classes range from I to IV, with higher values indicating more disabling pain due to angina.

EF, left ventricular ejection fraction; CTO, chronic total occlusion; RCA, right coronary artery; LAD, left anterior descending arteries; RPD, right posterior descending artery; Cx, circumflex artery; Mg, marginal branch circumflex artery.

Analyzing the data from the operation, on average 2.2 grafts were performed in CTO-RCA group and 2.4 grafts were implanted in nonCTO-RCA group (*p* = 0.003). Grafts to the RCA or RPD were performed in 40.3% of patients in the CTO-RCA group and in 81% patients in the nonCTO-RCA group (*p* = 0.001).

The overall mortality in the study group was 3.8%. The mortality from any cause of death or from cardiac cause did not differ between CTO-RCA and nonCTO-RCA group (Table 2). Likewise, post-operative atrial fibrillation, sternal wound complication and major bleeding at 30 days postoperative did not differ between CTO-RCA and nonCTO-RCA group. Incidence of MACCE was significantly more frequent in the CTO-RCA group (15.7% vs. 4%, *p* = 0.001). Importantly, we did not observe any differences in MACCE between patients with successful and non-successful implantation of bypass graft to RCA or RPD (6.73% vs. 11.32%; *p* = 0.324). In multivariate analysis, preoperative CTO in RCA or RPD (OR 7.9 CI [1.434–43.045]; *p* = 0.02) and co-existing peripheral artery disease (PAD) (OR 18.8 CI [3.451–101.833]; *p* < 0.01) were significant risk factors that increased the probability of post-operative MACCE (Table 3).

**Table 2:** 30-day clinical outcomes and adverse events.

Variable	CTO RCA/RPD (*N* = 57)	Non-CTO RCA/RPD (*N* = 99)	Hazard ratio relative risk (95% CI)	*p* value
	Number (percent)			
**Clinical outcome**				
Primary outcome: death from any cause	4 (7)	2 (2)	3.4 (0.6–18)	0.14
Primary outcome: death from cardiac cause	2 (3.5)	2 (2)	1.7 (0.25–11)	0.57
Composite end-point: death from any cause, myocardial infarction, cerebrovascular ischemia	9 (15.7)	4 (4)	3.9 (1.2–12.0)	0.01
Myocardial infarction	3 (5.2)	2 (2)	2.6 (0.4–15.1)	0.28
Cerebrovascular ischemia	2 (3.5)	0	3.2 (0.6–16)	0.22
**Adverse event**				
Atrial fibrillation	3 (5.2)	6 (6)	0.8 (0.2–3.3)	0.8
Sternal wound complication	0 (0)	7 (7)	0.1 (0.0–1.9)	0.13
Major bleeding	0 (0)	11 (11)	0.07 (0.0–1.4)	0.07

**Note:**

Hazard ratios are presented for clinical outcomes, and relative risks for adverse events. Hazard ratios use the non-CTO RCA/RPD group as the control.

**Table 3:** Multivariate logistic regression presenting risk factors for post-operative MACCE (*, 95% CI).

Variable	Hazard ratio relative risk (95% CI)*	*p* value
CTO RCA/RPD	7.9 (1.434–43.045)	0.02
PAD	18.8 (3.451–101.833)	<0.01
Age—yrs	1.1 (0.985–1.193)	0.10
Sex—female	0.5 (0.084–3.468)	0.52
Left ventricle ejection fraction <30%	1.6 (0.045–59.417)	0.79
Previous myocardial infarction	0.2 (0.023–1.003)	0.06
Previous stroke	1.9 (0.228–15.677)	0.554

**Note:**

*p* = 0.0001; Pseudo *R*^2^ = 0.2386.

## Discussion

The most accepted definition of complete revascularization (CR) is successful treatment of all major epicardial coronary vessels by either CABG or PCI (Strauss, Shuvy & Wijeysundera, 2014; Oshimaa et al., 2016). In SYNTAX trial (Ong & Serruys, 2006; Farooq et al., 2013), patients with total occlusion were less likely to achieve CR in the PCI and CABG arms (CR PCI: non-TO 59.8%, TO 34.3%, *p* = 0.001; CR CABG: non-TO 69.8%, TO 64.8%, *p* = 0.048).

In our study, significantly fewer grafts were implanted in the CTO-RCA group. This difference was particularly evident when analyzing the frequency of the RCA/RPD grafts. Two times more patients (81%) had a RCA/RPD grafts implanted in the nonCTO-RCA group as compared to the patients in CTO-RCA group (40.3%). Similar observations concerning the number of bypass grafts to the totally occluded RCA were reported by Rastan et al. (2009). The difference in bypass grafting to the RCA territory (CR 67.1 vs. incomplete revascularization (IR) 44.6%) was more obvious than to the circumflex territory (CR 87.0 vs. IR 73.6%).

Notably, a similar phenomenon of lower number of grafts to CTO arteries was reported in the Arterial Revascularization Therapies Study (van den Brand et al., 2002). A potential explanation for this finding might be that CTO in RCA is associated with more diffuse atherosclerosis, small vessel disease and in consequence is hindering the anastomosis of a bypass graft distally.

Coronary collaterals are frequently observed in CTO and vary in extent depending on the patient. Although rich collateral circulation to the CTO can potentially compete with the graft flow, its influence on graft patency is not well known. Furthermore, the relationship between graft failure and regional wall motion of the territory supplied by a CTO is unknown (Oshimaa et al., 2016).

Both more diffuse atherosclerosis and extent of collateral circulation may affect the perioperative results of CABG patients diagnosed with CTO. Based on randomized control studies, 30-day mortality after CABG ranges from 2.2% to 4.8% (Kowalewski et al., 2016; Deppe et al., 2016). In our study, the average mortality rate was 3.8% and was comparable to other studies. It should be emphasized that cardiac morbidity did not differ between patients with and without CTO in RCA. Basing on our results, we can conclude that the presence of CTO in the RCA does not affect 30-day total and cardiac mortality. Similarly, Rastan et al. (2009), based on the analysis of 8,806 consecutive patients with multivessel CAD, showed no major differences in post-operative complications and hospital mortality rate (3.3% for CR and 3.2% for IR; *p* = 0.520). In a CASS study (Bell et al., 1992) authors reported that 3,372 CABG patients with 3-vessel CAD who underwent operation between 1,974 and 1,979, had no significant difference in operative mortality. Likewise, Jones & Weintraub (1996) found similar consequences of IR regarding hospital and follow-up mortality in 2,857 patients with multivessel CAD. What is even more important, despite a statistically significantly lower total number of grafts and fewer RCA/RPD grafts in patients with CTO of RCA, IR had no significant effect on perioperative mortality rate.

In multivariate analysis we showed that co-existing PAD and CTO in RCA or RPD were significant risk factors increasing the probability of post-operative MACCE. Significantly higher MACCE incidence resulted from the observed difference in terms of MI rate and all-cause mortality between CTO-RCA and nonCRTO-RCA group. The effect of PAD on perioperative complications has been described in many studies. Logistic regression analysis showed that even after controlling for confounders, CABG patients with PAD still experienced more arrhythmias, neurological complications, pulmonary complications, low output and intraoperative complications. Notably, when comparing MACCE in patients with complete and IR in CTO-RCA group, we did not observe any significant differences. Although CR is recommended by the guidelines and patients with CR have better outcomes, our study showed that revascularization of CTO in RCA may be staged and performed by PCI during second procedure. Such staged procedure may have positive influence on long term survival and incidence of MACCE, however new studies are needed to confirm this theory.

## Limitations to the Study

Limitations of our study include its retrospective single-center observational design and small sample size, which could affect MACCE differences between complete and non-CR in patients with CTO in RCA. Finally, we have assessed only 30-day mortality so how CTO in RCA during CABG procedures affects long term mortality is yet to be shown.

## Conclusions

We have shown that preoperative presence of CTO of RCA is associated with smaller total number of grafts as well as grafts to RCA/RPD territory. This may be caused by the characteristics of RCA with CTO. Although CTO of RCA/RPD did not affect perioperative total or cardiac mortality it increased the risk of post-operative MACCE. If CTO is present in RCA, additional screening for PAD should be performed as those two variables were predictors of MACCE in our study.

## Supplemental Information

10.7717/peerj.4909/supp-1 Supplemental Information 1 Dataset.

## Additional Information and Declarations

### Data Availability

The following information was supplied regarding data availability: The raw data are provided in a Supplemental File.
